# Development of Novel Nrf2/ARE Inducers Bearing Pyrazino[2,1-*a*]isoquinolin Scaffold with Potent In Vitro Efficacy and Enhanced Physicochemical Properties

**DOI:** 10.3390/molecules22091541

**Published:** 2017-09-13

**Authors:** Hongbin Dai, Qiong Jiao, Tian Liu, Qidong You, Zhengyu Jiang

**Affiliations:** 1 Jiangsu Hengrui Medicine Company, Kunlunshan Road, Lianyungang Eco & Tech Development Zone, Lianyungang 222047, China; hengruigy@163.com; 2 State Key Laboratory of Natural Medicines and Jiangsu Key Laboratory of Drug Design and Optimization, China Pharmaceutical University, Nanjing 210009, China; yhjyscpu@163.com (Q.J.); 18136484953@163.com (T.L.)

**Keywords:** Nrf2 activator, pyrido[1,2-*a*]pyrazin analogues, oxidative stress, physicochemical properties

## Abstract

Pyrazino[2,1-*a*]isoquinolin analogues were reported as potent activators of Nrf2/ARE signaling both in vitro and in vivo by our group. In this study, we simplified the ring system to investigate the functions of various parts of the pyrazino[2,1-*a*]isoquinolin scaffold. We proved that the tetrahydroisoquinoline was not essential for activity and the pyrido[1,2-*a*]pyrazin analogues 3b and 3g retained the cellular Nrf2/ARE activation activity. Besides, this simplification significantly enhanced water solubility and membrane permeability, indicating that these compounds are more favourable for the further development of therapeutic agents around Nrf2 activation.

## 1. Introduction

Nuclear factor erythroid 2-related factor 2 (Nrf2) is a key transcription factor which plays a central role in inducible expression of many cytoprotective proteins in response to oxidative and electrophilic stress [1,2]. Under normal condition, Nrf2 is constantly degraded via the ubiquitin-proteasome in Kelch-like ECH-associated protein 1 (Keap1)-dependent manner in the cell cytoplasm [3,4]. Under oxidative or electrophilic stressed conditions, the thiols of Keap1 are modified by oxidization or alkylation resulting in the change of Keap1 conformation and the cease of Nrf2 ubiquitination and degradation. Then, the newly synthesized Nrf2 can translocate into nuclei to induce the transcription of target genes with Maf proteins [4,5,6]. These target genes all contain the enhancer sequence of antioxidant response element (ARE) in their promoter regulatory region, which are involved in glutathione synthesis, elimination of reaction oxygen species (ROS), detoxification of xenobiotics, and drug transport [7,8].

**Figure 1:**
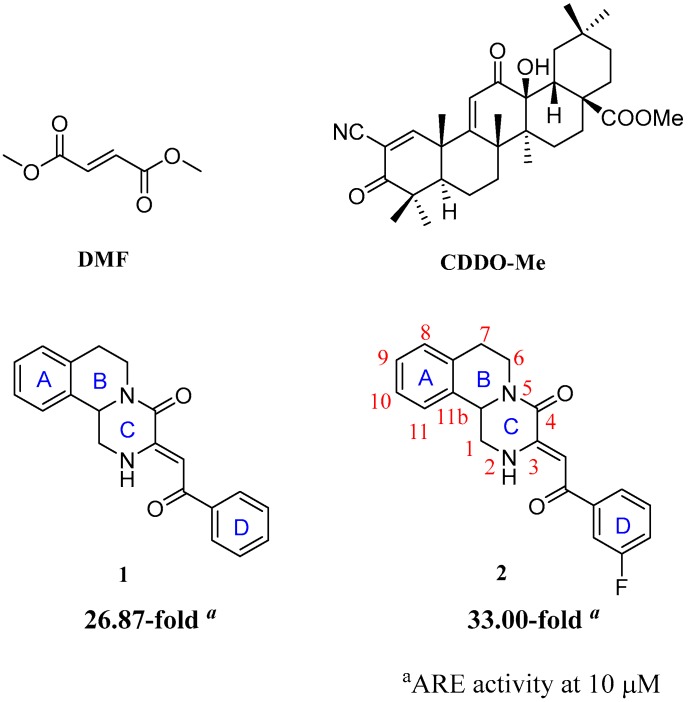
Structures of Dimethyl fumarate (DMF), synthetic triterpenoid bardoxolone methyl (CDDO-Me), **1** and **2**.

Therefore, identifying Nrf2 activators has been regarded as a potential strategy in the development of antioxidant, anti-inflammatory, or chemoprevention agents [9,10]. However, until now the progress in druglike Nrf2/ARE inducers discovery is limited [11]. The most successful Nrf2/ARE inducers are traditional electrophilic Nrf2 activators, which may mimic this endogenous process of Nrf2 by Keap1 thiols modification [12,13]. For example, Dimethyl fumarate (DMF) and synthetic triterpenoid bardoxolone methyl (CDDO-Me) are two well-known Nrf2 inducers (Figure 1) [14,15]. DMF has been approved by the FDA as a first-line oral drug for relapsing forms of multiple sclerosis (MS).

Our group previously discovered a novel small-molecule Nrf2/ARE inducer **1** by screening the in-house database using luciferase reporter assay (26.87-fold induction of ARE at 10 μM) [16]. This compound could induce the nuclear translocation of Nrf2 and the expression of NAD(P)H/quinone oxidoreductase 1 (NQO1) and heme oxygenase-1 (HO-1) in human colorectal cancer cells (HCT116), and significantly inhibit the development of colorectal adenomas in the azoxymethane (AOM)-dextran sodium sulfate (DSS) mouse model. For a preliminary structure-activity relationship (SAR) investigation, a more potent compound **2** with –F substitution at meta position of D ring was identified (33.00-fold induction of ARE at 10 μM). However, in later studies, **2** showed disadvantages in physicochemical properties, especially poor water solubility (0.022 μg/μL, pH 7.4) and poor membrane permeability (11.680 × 10^−6^ cm/s, pH 7.4). The poor physicochemical properties may be from the complex ring systems and the rigid backbone. Thus, we conducted a forward investigation of the structure-activity and structure-property relationships of the ring systems to improve the drug-like properties.

**2** contains four rings (A, B, C, D) and a, β-unsaturated ketone. According to DMF and CDDO-Me [17], a, β-unsaturated ketone is essential for Nrf2/ARE induction. Thus, in this study, we retained a, β-unsaturated ketone and mainly focused our efforts on investigating the compounds with polar side chains at D ring and compounds without A ring or D ring, for finding novel Nrf2/ARE inducers with comparable in vitro efficacy and enhanced physicochemical properties.

Herein, as a part of our ongoing program, we synthesized 24 compounds in three series totally and determined the induction folds of ARE activity in HepG2-ARE-C8 cell and IC_50_ values in HCT116 cells. First, we retained the backbone and introduced polar group substitutions at the benzene ring D by amino group (**1a**–**1e**). The results showed that introduction of the polar groups at the D ring was unfavorable to ARE induction activity. Secondly, various replacements of benzene ring D resulted in much weaker Nrf2 activators, **2a**–**2i**. The results showed that the benzene ring D is a key driver of the Nrf2/ARE induction effects. Finally, the removal of benzene ring A resulted in **3a**–**3j**, containing the pyrido[1,2-*a*]pyrazin scaffolds. **3b** and **3g** had comparable effects on ARE induction at 10 μM. 3g could elevate protein levels of Nrf2 and Nrf2-regulated genes, NAD(P)H: quinone oxidoreductase-1 (NQO1). Subsequently, physicochemical properties of **1e**, **2c**, **3a**, **3b** and **3g** were determined for further analysis, including p*K*a, partition coefficient (log *D*, pH 7.4), permeability (Pe) and water solubility (solubility, pH 7.4). They showed higher permeability and water solubility than **2**. Taken together, **3b** and **3g** showed improved physicochemical properties with comparable inducing activities of Nrf2/ARE at the cellular level. Thus, **3b** and **3g** were deserved further optimization with the aim to obtain therapeutic agents through Keap1-Nrf2-ARE pathways.

## 2. Results and Discussions

### 2.1. Chemistry

**Scheme 1:**
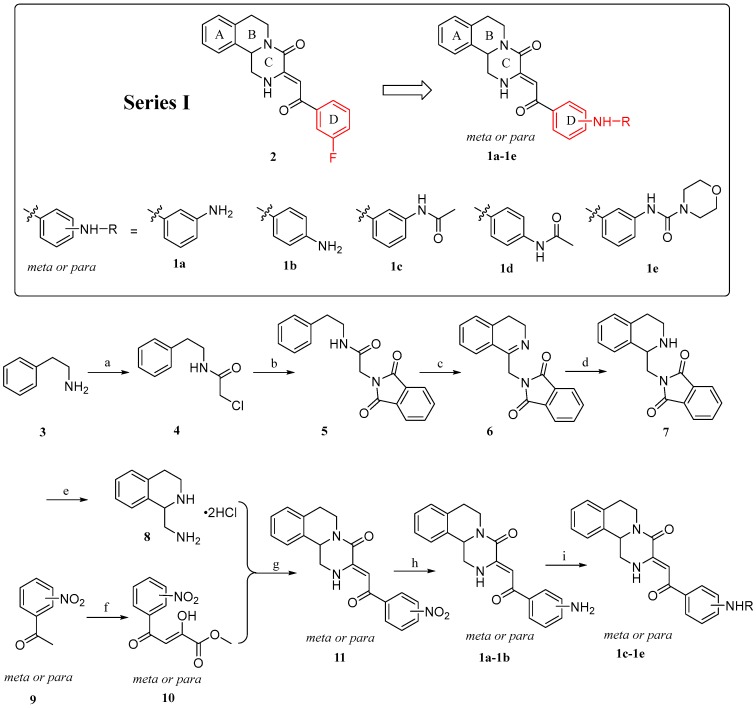
Structures and synthesis of **1a**–**1e**. Reagents and conditions: (a) chloracetyl chloride, NaHCO_3_, DCM, 0–10 °C, 2 h; (b) phthalimide potassium, DMF, 90 °C, 12 h; (c) P_2_O_5_,CH_3_CN, reflux, 2 h; (d) NaBH_4_, CH_3_OH, 25 °C, 3 h; (e) NaBH_4_, i-PrOH/H_2_O (6:1), AcOH, 80 °C, 2 h; (f) diethyl oxalate, NaOMe, MeOH, reflux, 4 h; (g) NaHCO_3_, EtOH, H_2_O, AcOH, reflux, 2 h; (h) SnCl_2_·2H_2_O, EA, reflux, 12 h; (i) acid anhydride or acid chloride, DMF, 30–90 °C, 4–24 h.

**1a**–**1e** were synthesized according to the protocol outlined in Scheme 1. Intermediate **8** was furnished via acylated, alkylated, cyclizated, reducted, deprotected from phenethylamine. At the same time, *meta* or *para* amino substituted acetophenone with diethyl oxalate via Claisen condensation gave the respective **10**. Subsequent reaction of **8** with **10** afforded the corresponding products **1a**–**1b**. Acylation of **1a**–**1b** resulted in **1c**–**1e**.

**Scheme 2:**
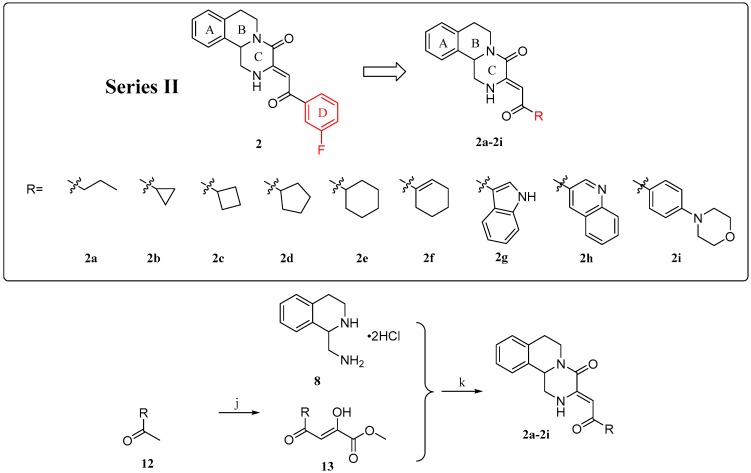
Structures and synthesis of **2a**–**2i**. Reagents and conditions: (j) diethyl oxalate, NaOMe, MeOH, reflux, 4 h; (k) NaHCO_3_, EtOH, H_2_O, AcOH, reflux, 2 h.

The synthesis routes of **2a**–**2i** was similar with those of **1a**–**1e** from the same intermediate **8** (Scheme 2). Acetylated materials **9** with diethyl oxalate via Claisen condensation gave the respective **10**. Subsequent reaction of **8** with **10** afforded the corresponding products **2a**–**2i**.

**Scheme 3:**
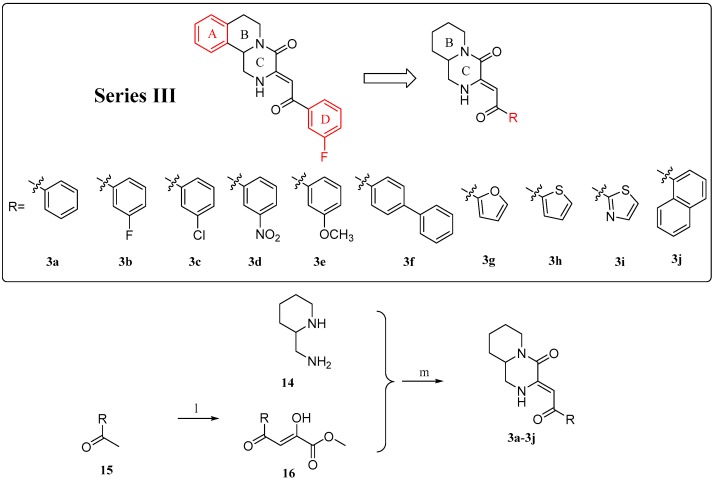
Structures and synthesis of **3a**–**3j**. Reagents and conditions: (l) diethyl oxalate, NaOMe, MeOH, reflux, 4 h; (m) NaHCO_3_, EtOH, H_2_O, AcOH, reflux, 2 h.

**3a**–**3j** were synthesized according to the protocol outlined in Scheme 3. Acetylated materials **15** with diethyl oxalate via Claisen condensation gave the respective **16**. Subsequent reaction of **16** with **14** afforded the corresponding products **3a**–**3j**.

### 2.2. ARE Luciferase Reporter Assay and Cytotoxicity Evaluation

All the derivatives were evaluated for their ability to induce ARE in HepG2-ARE-C8 cell (HepG2 cells stably transfected with a luciferase reporter) by ARE luciferase reporter assay, and they were compared to the lead compound **2**. At the same time, cytotoxicity studies were performed by the MTT assay against the HCT116 cells. The results of cytotoxic activity in vitro were expressed as the IC_50_ [18]. Because those compounds were designed for chemoprevention, they should not be cytotoxic. In general, as shown in Table 1, it was found that all of the compounds showed moderate cytotoxicity against the HCT116 cell line (IC_50_ ≥ 92.36 μM).

The previously reported ARE induction fold and IC_50_ value for **2** were 33.00-fold (10 μM) and >100 μM, respectively. The present measured ARE induction fold and IC_50_ value for **2** were 31.17-fold (10 μM) and 165.1 μM, respectively, consistent to the reported data. It is worth mentioning that **2** dose dependently induced ARE below 10 μM as usual, but reversed at 50 μM and 100 μM (Table 1). The reversal at high concentration may be due to the cytotoxicity at the high administrated concentration. Thus, we chose the induction folds at 10 μM to evaluate the induction activity in the ensuing discussion. Besides, in comparison, the moderate Nrf2 activator *tert*-butylhydroquinone (tBHQ) showed 10.79-fold induction at 100 μM (Table 1).

In general, 24 compounds showed quite different ARE induction activities in the ARE luciferase reporter assay. In the first series, **1a**–**1e** showed the dramatic decrease in activity, indicated that the polar group cannot replace the –F on D ring. **1e,** with morpholinecarbonyl is the most potent one in this series without a reversal at a high concentration.

In the next series, considering the rigid ring systems may lead to the unacceptable physicochemical properties, various replacements of the planar aromatic π system D ring were chosen as the next optimization sites. Replacements of rigid D ring with more flexible n-propyl, cyclopropyl, cyclobutyl, cyclopentyl, cyclohexyl, and cyclohexene resulted in **2a**–**2f**. The luciferase reporter assay results showed that almost all of them showed a dramatic decrease in activity, indicating the necessary of the benzene ring. On the other hand, much larger rigid structures, the indole ring **2g**, quinoline ring **2h** and phenylmorpholine **2i** were synthesized for further SAR studies. Induction fold of those three candidates **2g**–**2i** were even smaller than that of six previous candidates **2a**–**2f**. In this series, **2c** showed some interesting characters. It was the most effective in ARE induction in this series, and its ARE induction effect was dose-dependent for the whole concentration ranges. **2i** with phenylmorpholine group showed dramatically decreased activity followed the trend of polar group substitution at D ring as described above. In summary, the lower right corner of the structure is the key driver of the Nrf2/ARE induction effects, and the benzene ring with 3-F substitution was still the best so far.

In the last series, the upper left corner benzene ring A were chosen as the next optimization. Removal of A ring in **2** gave **3b**, and it had a comparable effect on ARE induction at 10 μM, which indicated that A ring was not the determinant for activity. Secondly, replacements of F with H, electron-donating groups and electron-withdrawing groups gave **3a**, **3c**, **3d**, **3e** and **3f**. Generally, none of them are comparable with **3b** in activity. It was consistent with that the benzene ring with 3-F substitution was the best. **3e** and **3f** with an electron-donating group were not preferred for ARE induction activity, whereas the **3c** and **3d** with an electron-withdrawing group exhibited better performance. This observation indicated that electron-withdrawing groups in the Michael acceptor may be beneficial for the covalent modification with Keap1 cysteine residues.

According to above research, replacements of D ring with both cycloalkanes and fused heterocycles (indole and quinolone) reduced activities. Thus, we focused on the replacements of single heterocycles and naphthalene in this series, and synthesized **3g**–**3j**. The replacement of D ring for naphthalene ring **3j** showed little effect on ARE induction activity, whereas the furan ring **3g**, thiophene ring **3h,** and thiazole ring **3i** showed increased activity. **3g** dose dependently increased ARE induction effects at all of the chosen concentrations, and it showed 27.01-fold induction of ARE at 10 μM and 79.74-fold at 100 μM. Also, the IC_50_ value of MTT assay showed that **3g** was not cytotoxic in the administrated concentration (the IC_50_ value of **3g** was more than 300 μM). Considering that both **3b** and **3g** remarkably activated ARE without A ring. We summarized that and benzene ring A was not critical for Nrf2/ARE inducing activity and it could be removed. The novel pyrido[1,2-*a*]pyrazin scaffold of the Nrf2 activator was obtained by removal of A ring of pyrazino[2,1-*a*]isoquinolin scaffold.

### 2.3. Western Blot Assay

**Figure 2:**
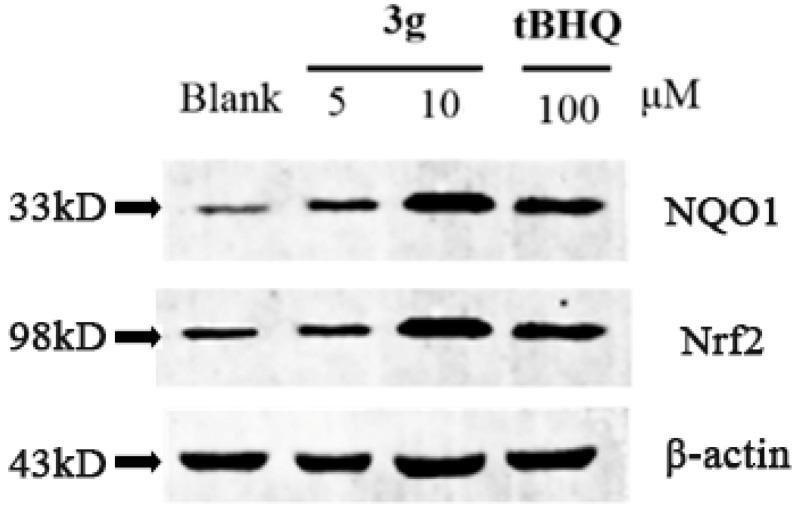
**3g** can elevate the protein level of Nrf2 and NQO1.

Compound **3g** was selected to evaluate its capacity to activate the expression of Nrf2-regulated NQO1 by measuring the protein levels in HCT116 via Western Blot [19]. In comparison, tBHQ induced expression of NQO1 at 100 μM. As shown in Figure 2, NQO1 were induced by **3g** in a concentration-dependent manner, and the effect is better than tBHQ at 100 μM. Moreover, **3g** also elevated the protein level of Nrf2 at 10 μM, and the up-regulation effect is more remarkable than tBHQ at 100 μM. Those data further confirmed that **3g** bearing pyrido[1,2-*a*]pyrazin scaffold could elevate both Nrf2 and downstream proteins of Nrf2 at the cellular level.

### 2.4. Physicochemical Properties

Then we evaluated the physicochemical properties of a selected series of our compounds. **1e** and **2c** were chosen as a representation of series I and series II, respectively. **3a**, **3b** and **3g** were selected from the most potent series III. All of them were evaluated with p*K*a, partition coefficient (log *D*, pH 7.4), membrane permeability (Pe, pH 7.4) and water solubility. The p*K*a and distribution coefficient (log *D*, pH 7.4) were determined according to the methods of Avdeef and Tsinman on a Gemini Profiler instrument (pION) by the “goldstandard” Avdeef-Bucher potentiometric titration method. Permeability (Pe) was determined by a standard parallel artificial membrane permeability assay (PAMPA by pION). Ketoprofen and propranolol were chosen as a control. Ketoprofen shows poor membrane permeability, while propranolol shows good membrane permeability.

As shown in Table 2, when compared with **2**, almost all of the new synthetic compounds had better membrane permeability and water solubility. This indicated that three strategies of introducing side chains at D ring and removing D ring or A ring were favorable for physicochemical properties. We found **3b** and **3g** significantly enhanced water solubility and membrane permeability with comparable in vitro inducing activities of Nrf2/ARE (32.97-fold and 27.01-fold induction of ARE at 10 μM). Whether **3b** and **3g** were better than 2 in vivo is still unrevealed and is worth being confirmed in further research.

## 3. Conclusions

**Figure 3:**
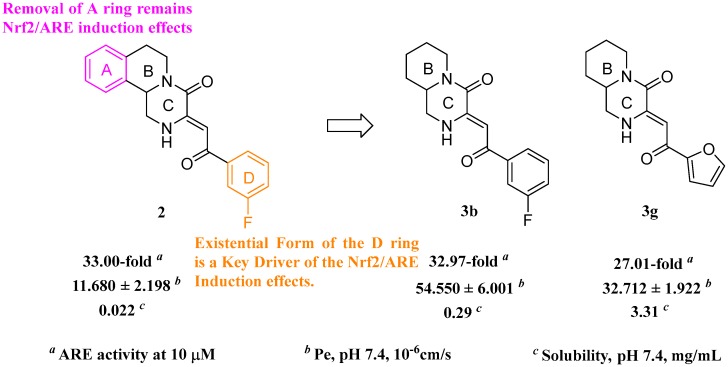
SAR results and developments of pyrazino[2,1-*a*]isoquinolin analogue **2** and pyrido[1,2-*a*]pyrazin analogues **3b** and **3g**.

In summary, three series of compounds were synthesized and evaluated as efficient activators for Nrf2 (Figure 3). The SAR studies revealed that the induction fold for ARE were not favored by the introduction of polar side chains at D ring. The lower right corner of the structure is a key driver of the Nrf2/ARE induction effects. Removal of A ring with proper lower right corner ring, such as 3-F benzene ring (**3b**) and furan ring (**3g**), can not only retain Nrf2/ARE induction activity, but also improve the physicochemical properties. The novel pyrido[1,2-*a*]pyrazin scaffold was obtained by removal of A ring of pyrazino[2,1-*a*]isoquinolin scaffold. Our Western Blot studies supported that the novel scaffold of pyrido[1,2-*a*]pyrazin structure still can elevate both Nrf2 and NQO1 at cellular level. These findings encourage further investigations of the novel pyrido[1,2-*a*]pyrazin analogues as Nrf2 chemoprevention agents.

## 4. Materials and Methods

### 4.1. Chemistry

#### 4.1.1. General Chemistry

All reagents were purchased from commercial sources and unless otherwise noted, were used without further purification. All of the synthesized compounds were characterized with ^1^H-NMR, ^13^C-NMR (300 MHz, Bruker, Karlsruhe, Germany), and HRMS (ESI+). Purity (≥95%) of target compounds was determined by the HPLC study performed on Agilent C18 (4.6 mm × 150 mm, 3.5 μm) column and peak detection at 254 nm under UV. ESI-mass and high resolution mass spectra (HRMS) were recorded on a Water Q-Tofmicro mass spectrometer. Analytical results are within 0.40% of the theoretical values.

#### 4.1.2. General Procedure for the Preparation of Compounds **1a**–**1e**

A solution of diethyl oxalate (1.82 mL, 13.40 mmol) and **9** (1.37 g, 8.32 mmol) in CH_3_OH (50 mL) was added dropwise to a solution of CH_3_ONa in CH_3_OH (3.33 mL of mol/L, 16.64 mmol), and the reaction was allowed to proceed at reflux for 4 h. After cooling to room temperature (RT), the mixture was poured into water (100 mL) and acidified with HCl (2 mL of 37% *w*/*v*). The precipitate was filtered to afford the respective product **10**. Yield 48–54%.

A solution of NaHCO_3_ (672 mg, 8 mmol) in water (4 mL) was added to a solution of the **8** (940 mg, 4 mmol) in ethanol (4 mL) and slightly heated until the evolution of gas ceased. A solution of the **10** (1004 mg, 4 mmol) in ethanol (4 mL) and glacial AcOH (2 mL) was then added and refluxed for 2 h. After cooling, the precipitated solid **11** was filtered off and recrystallized from ethanol. Yield 55–57%.

**11** (727 mg, 2 mmol) was dissolved in ethyl acetate (80 mL), after which SnCl_2_·2H_2_O (2.26 g, 10 mmol) was added. The reaction mixture was stirred at room temperature for 12 h. Ethyl acetate (200 mL) was added for the completely soluble mixture. Then, saturated sodium bicarbonate solution was added. The mixture was stirred for 15 min, followed by filtration. The organic extracts were concentrated in vacuo to give the product **1a**–**1b**. Yield 74–79%. **1a** or **1b** (100 mg, 0.3 mmol) was dissolved in DMF (2 mL), after which acetic anhydride (28.3 μL, 0.3 mmol) was added. The reaction mixture was stirred at room temperature for 4 h. The mixture was poured to water (50 mL). The precipitate was filtered and dried to give the product **1c**–**1d**. Yield 60–61%. **1a** (100 mg, 0.3 mmol) was dissolved in DMF (2 mL), after which 4-Morpholinecarbonyl chloride (35 μL, 0.3 mmol) and triethylamine (61 μL, 0.6 mmol) were added. The reaction mixture was stirred at room temperature for 4 h. The mixture was poured to water (50 mL). The precipitate was filtered and dried to give the crude product, which was purified through column chromatography over silica gel to give the product **1e**, Yield 34%.

*3-(2-(3-aminophenyl)-2-oxoethylidene)-1,2,3,6,7,11b-hexahydro-4H-pyrazino[2,1-a]isoquinolin-4-one* (**1a**) Yield 79%. Brown solid. m.p. 206–208 °C. ^1^H-NMR (300 MHz, DMSO-*d*_6_): δ 10.75 (d, *J* = 4.77 Hz, 1H), 7.33–7.24 (m, 4H), 7.13–7.08 (m, 2H), 7.01 (d, *J* = 7.71 Hz, 1H), 6.71–6.68 (m, 1H), 6.51 (s, 1H), 5.28 (s, 2H), 5.12–5.07 (m, 1H), 4.67–4.62 (m, 1H), 4.04–3.96 (m, 1H), 3.28 (t, *J* = 14.45 Hz, 1H), 3.19–2.81 (m, 3H). IR (cm^−1^, KBr film): 3448 (-NH), 1656 (-CON), 1613 (-CO). HRMS (ESI): calcd. for C_20_H_20_N_3_O_2_ [M + H]^+^ 334.155, found 334.1548, HPLC (80% methanol in water): *t*_R_ = 4.73 min, 95.39%.

*3-(2-(4-aminophenyl)-2-oxoethylidene)-1,2,3,6,7,11b-hexahydro-4H-pyrazino[2,1-a]isoquinolin-4-one* (**1b**) Yield 74%. Yellow solid. m.p. 238–240 °C. ^1^H-NMR (300 MHz, DMSO-*d*_6_): δ 10.57 (d, *J* = 4.89 Hz, 1H), 7.65 (d, *J* = 8.61 Hz, 2H), 7.35–7.26 (m, 4H), 6.59 (d, *J* = 8.61 Hz, 2H), 6.52 (s, 1H), 5.86 (s, 2H), 5.10–5.05 (m, 1H), 4.69–4.64 (m, 1H), 4.02–3.95 (m, 1H), 3.20 (t, *J* = 12.36 Hz, 1H), 3.02–2.88 (m, 3H). IR (cm^−1^, KBr film): 3442 (-NH), 1655 (-CON), 1594 (-CO). HRMS (ESI): calcd. for C_20_H_20_N_3_O_2_ [M + H]^+^ 334.155, found 334.1546, HPLC (80% methanol in water): *t*_R_ = 4.58 min, 95.20%.

*N-(3-(2-(4-oxo-1,6,7,11b-tetrahydro-2H-pyrazino[2,1-a]isoquinolin-3(4H)-ylidene)acetyl)phenyl)acetamide* (**1c**) Yield 61%. Yellow solid. m.p. 247–249 °C. ^1^H-NMR (300 MHz, DMSO-*d*_6_): δ 10.83 (d, *J* = 4.89 Hz, 1H), 10.12 (s, 1H), 8.09 (s, 1H), 7.81 (d, *J* = 8.97 Hz, 1H), 7.54 (d, *J* = 7.89 Hz, 1H), 7.40 (t, *J* = 7.89 Hz, 1H), 7.33–7.25 (m, 4H), 6.55 (s, 1H), 5.14–5.09 (m, 1H), 4.67–4.62 (m, 1H), 4.09–3.96 (m, 1H), 3.31–3.22 (m, 1H), 3.08–2.82 (m, 3H), 2.06 (s, 3H). IR (cm^−1^, KBr film): 3448 (-NH), 1668 (-CON), 1615 (-CO). HRMS (ESI): calcd. for C_22_H_22_N_3_O_3_ [M + H]^+^ 376.1656, found 376.1661, HPLC (80% methanol in water): *t*_R_ = 4.62 min, 95.11%.

*N-(4-(2-(4-oxo-1,6,7,11b-tetrahydro-2H-pyrazino[2,1-a]isoquinolin-3(4H)-ylidene)acetyl)phenyl)acetamide* (**1d**) Yield 60%. Yellow solid. m.p. 279–281 °C. ^1^H-NMR (300 MHz, DMSO-*d*_6_): δ 10.80 (d, *J* = 4.02 Hz, 1H), 10.22 (s, 1H), 7.88 (d, *J* = 8.70 Hz, 2H), 7.70 (d, *J* = 8.55 Hz, 2H), 7.33–7.28 (m, 4H), 6.59 (s, 1H), 5.14–5.09 (m, 1H), 4.68–4.60 (m, 1H), 4.05–4.01 (m, 1H), 3.26 (t, *J* = 12.80 Hz, 1H), 3.05–2.89 (m, 3H), 2.09 (s, 3H). IR (cm^−1^, KBr film): 3433 (-NH), 1692 (-CON), 1594 (-CO). HRMS (ESI): calcd. for C_22_H_22_N_3_O_3_ [M + H]^+^ 376.1656, found 376.1662. HPLC (80% methanol in water): *t*_R_ = 5.01 min, 95.26%.

*N-(3-(2-(4-oxo-1,6,7,11b-tetrahydro-2H-pyrazino[2,1-a]isoquinolin-3(4H)-ylidene)acetyl)phenyl)morpholine-4-carboxamide* (**1e**) Yield 34%. Yellow solid. m.p. 228–230 °C. ^1^H-NMR (300 MHz, DMSO-*d*_6_): δ 10.85 (d, *J* = 4.89 Hz, 1H), 8.77 (s, 1H), 8.02 (s, 1H), 7.73 (d, *J* = 7.95 Hz, 1H), 7.49 (d, *J* = 7.74 Hz, 1H), 7.39–7.27 (m, 5H), 6.58 (s, 1H), 5.16–5.11 (m, 1H), 4.69–4.64 (m, 1H), 4.08–4.01 (m, 1H), 3.64 (t, *J* = 4.67 Hz, 4H), 3.48–3.45 (m, 4H), 3.28 (t, *J* = 12.69 Hz, 1H), 3.04-2.85 (m, 3H). IR (cm^−1^, KBr film): 3365 (-NH), 1655 (-CON), 1614 (-CO). HRMS (ESI): calcd. for C_25_H_26_N_4_NaO_4_ [M + Na]^+^ 469.1846, found 469.1843, HPLC (80% methanol in water): *t*_R_ = 6.03 min, 98.42%.

#### 4.1.3. General Procedure for the Preparation of Compounds **2a**–**2i**

A solution of diethyl oxalate (1.82 mL, 13.40 mmol) and **12** (8.32 mmol) in CH_3_OH (50 mL) was added dropwise to a solution of CH_3_ONa in CH_3_OH (3.33 mL of mol/L, 16.64 mmol), and the reaction was allowed to proceed at reflux for 4 h. After cooling to RT, the mixture was poured into water (100 mL) and acidified with HCl (2 mL of 37% *w*/*v*). The precipitate was filtered to afford the respective product **13**. The product was ready for the next step without the further purification.

A solution of NaHCO_3_ (336 mg, 4 mmol) in water (3 mL) was added to a solution of the **8** (470 mg, 2 mmol) in ethanol (2 mL) and slightly heated until the evolution of gas ceased. A solution of the **13** (2 mmol) in ethanol (2 mL) and glacial AcOH (1 mL) was then added and refluxed for 2 h. After cooling, the precipitated solid **2a**–**2i** was filtered off and recrystallized from ethanol. Yield 55–82%.

*3-(2-oxopentylidene)-1,2,3,6,7,11b-hexahydro-4H-pyrazino[2,1-a]isoquinolin-4-one* (**2a**) Yield 67%. Yellow solid. m.p. 158–160 °C. ^1^H-NMR (300 MHz, DMSO-*d*_6_): δ 10.28 (d, *J* = 5.46 Hz, 1H), 7.30–7.25 (m, 4H), 5.85 (s, 1H), 5.08–5.02 (m, 1H), 4.65–4.57 (m, 1H), 3.98–3.90 (m, 1H), 3.16 (t, *J* = 12.44 Hz, 1H), 2.99–2.84 (m, 3H), 2.33 (t, *J* = 7.32 Hz, 2H), 1.61–1.49 (m, 2H), 0.88 (t, *J* = 7.38 Hz, 3H). IR (cm^−1^, KBr film): 3416 (-NH), 1665 (-CON), 1614 (-CO). HRMS (ESI): calcd. for C_17_H_20_N_2_NaO_2_ [M + Na]^+^ 307.1417, found 307.1416, HPLC (80% methanol in water): *t*_R_ = 6.40 min, 98.39%.

*3-(2-cyclopropyl-2-oxoethylidene)-1,2,3,6,7,11b-hexahydro-4H-pyrazino[2,1-a]isoquinolin-4-one* (**2b**) Yield 57%. Yellow solid. m.p. 173–175 °C. ^1^H-NMR (300 MHz, DMSO-*d*_6_): δ 10.21 (d, *J* = 4.71 Hz, 1H), 7.28–7.24 (m, 4H), 6.00 (s, 1H), 5.05–5.00 (m, 1H), 4.63–4.58 (m, 1H), 3.93–3.86 (m, 1H), 3.13 (t, *J* = 12.71 Hz, 1H), 2.98–2.79 (m, 3H), 2.02–1.88 (m, 1H), 0.78–0.75 (m, 4H). IR (cm^−1^, KBr film): 3416 (-NH), 1664 (-CON), 1612 (-CO). HRMS (ESI): calcd. for C_17_H_19_N_2_O_2_ [M + H]^+^ 283.1441, found 283.1434, HPLC (80% methanol in water): *t*_R_ = 5.69 min, 98.78%.

*3-(2-cyclobutyl-2-oxoethylidene)-1,2,3,6,7,11b-hexahydro-4H-pyrazino[2,1-a]isoquinolin-4-one* (**2c**) Yield 68%. Yellow solid. m.p. 172–174 °C. ^1^H-NMR (300 MHz, DMSO-*d*_6_): δ 10.24 (d, *J* = 4.92 Hz, 1H), 7.28–7.24 (m, 4H), 5.75 (s, 1H), 5.06–5.00 (m, 1H), 4.62–4.57 (m, 1H), 3.97–3.90 (m, 1H), 3.27–3.11 (m, 2H), 2.97–2.83 (m, 3H), 2.14–2.07 (m, 4H), 2.04–1.87 (m, 1H), 1.85–1.67 (m, 1H). IR (cm^−1^, KBr film): 3448 (-NH), 1663 (-CON), 1617 (-CO). HRMS (ESI): calcd. for C_18_H_21_N_2_O_2_ [M + H]^+^ 297.1598, found 297.1600, HPLC (80% methanol in water): *t*_R_ = 6.63 min, 95.01%.

*3-(2-cyclopentyl-2-oxoethylidene)-1,2,3,6,7,11b-hexahydro-4H-pyrazino[2,1-a]isoquinolin-4-one* (**2d**) Yield 67%. Yellow solid. m.p. 185–187 °C. ^1^H-NMR (300 MHz, DMSO-*d*_6_): δ 10.22 (d, *J* = 4.89 Hz, 1H), 7.28–7.23 (m, 4H), 5.86 (s, 1H), 5.06–5.00 (m, 1H), 4.63–4.57 (m, 1H), 3.95–3.88 (m, 1H), 3.14 (t, *J* = 12.45 Hz, 1H), 2.97–2.78 (m, 4H), 1.75–1.50 (m, 8H). IR (cm^−1^, KBr film): 3448 (-NH), 1664 (-CON), 1614 (-CO). HRMS (ESI): calcd. for C_19_H_23_N_2_O_2_ [M + H]^+^ 311.1754, found 311.1753, HPLC (80% methanol in water): *t*_R_ = 8.07 min, 98.99%.

*3-(2-cyclohexyl-2-oxoethylidene)-1,2,3,6,7,11b-hexahydro-4H-pyrazino[2,1-a]isoquinolin-4-one* (**2e**) Yield 61%. Yellow solid. m.p. 203–205 °C. ^1^H-NMR (300 MHz, DMSO-*d*_6_): δ 10.30 (d, *J* = 4.95 Hz, 1H), 7.28–7.24 (m, 4H), 5.86 (s, 1H), 5.06–5.00 (m, 1H), 4.62–4.55 (m, 1H), 3.95–3.88 (m, 1H), 3.15 (t, *J* = 12.50 Hz, 1H), 2.97–2.80 (m, 3H), 2.32–2.24 (m, 1H), 1.70–1.60 (m, 5H), 1.34–1.16 (m, 5H). IR (cm^−1^, KBr film): 3448 (-NH), 1662 (-CON), 1616 (-CO). HRMS (ESI): calcd. for C_20_H_25_N_2_O_2_ [M + H]^+^ 325.1911, found 325.1905, HPLC (80% methanol in water): *t*_R_ = 9.90 min, 98.61%.

*3-(2-(cyclohex-1-en-1-yl)-2-oxoethylidene)-1,2,3,6,7,11b-hexahydro-4H-pyrazino[2,1-a]isoquinolin-4-one* (**2f**) Yield 55%. Yellow solid. m.p. 180–182 °C. ^1^H-NMR (300 MHz, DMSO-*d*_6_): δ 10.47 (d, *J* = 5.13 Hz, 1H), 7.29–7.23 (m, 4H), 6.71 (s, 1H), 6.23 (s, 1H), 5.06–5.01 (m, 1H), 4.64–4.56 (m, 1H), 3.97–3.90 (m, 1H), 3.16 (t, *J* = 12.35 Hz, 1H), 2.98–2.80 (m, 3H), 2.30–2.14 (m, 4H), 1.59–1.57 (m, 4H). IR (cm^−1^, KBr film): 3448 (-NH), 1658 (-CON), 1608 (-CO). HRMS (ESI): calcd. for C_20_H_23_N_2_O_2_ [M + H]^+^ 323.1754, found 323.1760, HPLC (80% methanol in water): *t*_R_ = 9.04 min, 98.19%.

*3-(2-(1H-indol-3-yl)-2-oxoethylidene)-1,2,3,6,7,11b-hexahydro-4H-pyrazino[2,1-a]isoquinolin-4-one* (**2g**) Yield 59%. Yellow solid. m.p. 270–272 °C. ^1^H-NMR (300 MHz, DMSO-*d*_6_): δ 11.79 (s, 1H), 10.38 (d, *J* = 4.74 Hz, 1H), 8.28 (d, *J* = 6.93 Hz, 1H), 8.18 (d, *J* = 2.10 Hz, 1H), 7.43 (d, *J* = 8.37 Hz, 1H), 7.34–7.26 (m, 4H), 7.20–7.11 (m, 2H), 6.50 (s, 1H), 5.09–5.05 (m, 1H), 4.68–4.65 (m, 1H), 4.00–3.94 (m, 1H), 3.19 (t, *J* = 12.54 Hz, 1H), 3.00–2.87 (m, 3H). IR (cm^−1^, KBr film): 3448 (-NH), 1644 (-CON), 1605 (-CO). HRMS (ESI): calcd. for C_22_H_20_N_3_O_2_ [M + H]^+^ 358.1550, found 358.1546, HPLC (80% methanol in water): *t*_R_ = 6.41 min, 95.67%.

*3-(2-oxo-2-(quinolin-3-yl)ethylidene)-1,2,3,6,7,11b-hexahydro-4H-pyrazino[2,1-a]isoquinolin-4-one* (**2h**) Yield 82%. Yellow solid. m.p. 245–247 °C. ^1^H-NMR (300 MHz, DMSO-*d*_6_): δ 11.00 (d, *J* = 4.74 Hz, 1H), 9.34 (d, *J* = 2.10 Hz, 1H), 8.94 (d, *J* = 1.86 Hz, 1H), 8.22 (d, *J* = 7.74 Hz, 1H), 8.08 (d, *J* = 8.04 Hz, 1H), 7.89–7.84 (m, 1H), 7.69 (t, *J* = 7.47 Hz, 1H), 7.35–7.26 (m, 4H), 6.77 (s, 1H), 5.18–5.13 (m, 1H), 4.70–4.65 (m, 1H), 4.12–4.04 (m, 1H), 3.37–3.28 (m, 1H), 3.06–2.84 (m, 3H). IR (cm^−1^, KBr film): 3424 (-NH), 1664 (-CON), 1616 (-CO). HRMS (ESI): calcd. for C_23_H_20_N_3_O_2_ [M + H]^+^ 370.1550, found 370.1550, HPLC (80% methanol in water): *t*_R_ = 7.04 min, 96.28%.

*3-(2-(4-morpholinophenyl)-2-oxoethylidene)-1,2,3,6,7,11b-hexahydro-4H-pyrazino[2,1-a]isoquinolin-4-one* (**2i**) Yield 57%. Yellow solid. m.p. 235–237 °C. ^1^H-NMR (300 MHz, DMSO-*d*_6_): δ 10.67 (d, *J* = 4.77 Hz, 1H), 7.79 (d, *J* = 8.94 Hz, 2H), 7.33–7.24 (m, 4H), 6.99 (d, *J* = 9.03 Hz, 2H), 6.55 (s, 1H), 5.11–5.05 (m, 1H), 4.67–4.62 (m, 1H), 4.03–3.95 (m, 1H), 3.73 (t, *J* = 4.76 Hz, 4H), 3.26–3.17 (m, 5H), 3.01–2.86 (m, 3H). IR (cm^−1^, KBr film): 3442 (-NH), 1663 (-CON), 1597 (-CO). HRMS (ESI): calcd. for C_24_H_26_N_3_O_3_ [M + H]^+^ 404.1969, found 404.1973, HPLC (80% methanol in water): *t*_R_ = 6.58 min, 95.13%.

#### 4.1.4. General Procedure for the Preparation of Compounds **3a**–**3j**

A solution of diethyl oxalate (1.82 mL, 13.40 mmol) and **15** (8.32 mmol) in CH_3_OH (50 mL) was added dropwise to a solution of CH_3_ONa in CH_3_OH (3.33 mL of mol/L, 16.64 mmol), and the reaction was allowed to proceed at reflux for 4 h. After cooling to RT, the mixture was poured into water (100 mL) and acidified with HCl (2 mL of 37% *w*/*v*). The precipitate was filtered to afford the respective product **16**. The product was ready for the next step without the further purification.

**14** (242 μL, 2 mmol) and **16** (2 mmol) were was dissolved in ethanol (10 mL), after which glacial AcOH (1 mL) was added. The reaction was allowed to proceed at reflux for 2 h. After cooling, the precipitated solid **3a**–**3j** was filtered off and recrystallized from ethanol. Yield 45.79–69.66%.

*3-(2-oxo-2-phenylethylidene)octahydro-4H-pyrido[1,2-a]pyrazin-4-one* (**3a**) Yield 59%. Yellow solid. m.p. 164–166 °C. ^1^H-NMR (300 MHz, DMSO-*d*_6_): δ 10.64 (s, 1H), 7.86 (d, *J* = 1.32 Hz, 2H), 7.84–7.44 (m, 3H), 6.59 (s, 1H), 4.38 (d, *J* = 13.32 Hz, 1H), 3.64–3.58 (m, 2H), 3.33–3.21 (m, 1H), 2.75–2.66 (m, 1H), 1.79–1.72 (m, 3H), 1.49–1.30 (m, 3H). IR (cm^−1^, KBr film): 3422 (-NH), 1658 (-CON), 1611 (-CO). HRMS (ESI): calcd. for C_16_H_19_N_2_O_2_ [M + H]^+^ 271.1441, found 271.1439, HPLC (80% methanol in water): *t*_R_ = 7.63 min, 99.31%.

*3-(2-(3-fluorophenyl)-2-oxoethylidene)octahydro-4H-pyrido[1,2-a]pyrazin-4-one* (**3b**) Yield 59%. Yellow solid. m.p. 156–158 °C. ^1^H-NMR (300 MHz, DMSO-*d*_6_): δ 10.72 (s, 1H), 7.72 (d, *J* = 7.83 Hz, 1H), 7.61–7.51 (m, 2H), 7.42–7.35 (m, 1H), 6.58 (s, 1H), 4.40 (d, *J* = 13.44 Hz, 1H), 3.69–3.62 (m, 2H), 3.34–3.24 (m, 1H), 2.78–2.70 (m, 1H), 1.82–1.74 (m, 3H), 1.44–1.25 (m, 3H). IR (cm^−1^, KBr film): 3455 (-NH), 1657 (-CON), 1613 (-CO). HRMS (ESI): calcd. for C_16_H_18_FN_2_O_2_ [M + H]^+^ 289.1347, found 289.1348, HPLC (80% methanol in water): *t*_R_ = 7.80 min, 98.74%.

*3-(2-(3-chlorophenyl)-2-oxoethylidene)octahydro-4H-pyrido[1,2-a]pyrazin-4-one* (**3c**) Yield 66%. Yellow solid. m.p. 157–159 °C. ^1^H-NMR (300 MHz, DMSO-*d*_6_): δ 10.71 (s, 1H), 7.84–7.82 (m, 2H), 7.63–7.50 (m, 2H), 6.56 (s, 1H), 4.40 (d, *J* = 13.29 Hz, 1H), 3.70–3.62 (m, 2H), 3.30–3.25 (m, 1H), 2.78–2.70 (m, 1H), 1.82–1.74 (m, 3H), 1.52–1.40 (m, 3H). IR (cm^−1^, KBr film): 3416 (-NH), 1672 (-CON), 1605 (-CO). HRMS (ESI): calcd. for C_16_H_18_ClN_2_O_2_ [M + H]^+^ 305.1051, found 305.1055, HPLC (80% methanol in water): *t*_R_ = 8.26 min, 98.81%.

*3-(2-(3-nitrophenyl)-2-oxoethylidene)octahydro-4H-pyrido[1,2-a]pyrazin-4-one* (**3d**) Yield 61%. Yellow solid. m.p. 174–176 °C. ^1^H-NMR (300 MHz, DMSO-*d*_6_): δ 10.78 (s, 1H), 8.55 (s, 1H), 8.38–8.35 (m, 1H), 8.30 (d, *J* = 7.80 Hz, 1H), 7.77 (t, *J* = 7.87 Hz, 1H), 6.61 (s, 1H), 4.37 (d, *J* = 13.29 Hz, 1H), 3.68–3.64 (m, 2H), 3.32 (m, 1H), 2.77–2.68 (m, 1H), 1.80–1.77 (m, 3H), 1.49–1.14 (m, 3H). IR (cm^−1^, KBr film): 3449 (-NH), 1666 (-CON), 1614 (-CO). HRMS (ESI): calcd. for C_16_H_18_N_3_O_4_ [M + H]^+^ 316.1292, found 316.1293, HPLC (80% methanol in water): *t*_R_ = 6.93 min, 95.92%.

*3-(2-(3-methoxyphenyl)-2-oxoethylidene)octahydro-4H-pyrido[1,2-a]pyrazin-4-one* (**3e**) Yield 48%. Yellow solid. m.p. 133–135 °C. ^1^H-NMR (300 MHz, DMSO-*d*_6_): δ 10.66 (s, 1H), 7.46–7.35 (m, 3H), 7.13–7.10 (m, 1H), 6.58 (s, 1H), 4.40 (d, *J* = 13.32 Hz, 1H), 3.82 (s, 3H), 3.66–3.60 (m, 2H), 3.28–3.23 (m, 1H), 2.78–2.69 (m, 1H), 1.81–1.78 (m, 3H), 1.48–1.25 (m, 3H). IR (cm^−1^, KBr film): 3422 (-NH), 1658 (-CON), 1608 (-CO). HRMS (ESI): calcd. for C_17_H_21_N_2_O_3_ [M + H]^+^ 301.1547, found 301.1534, HPLC (80% methanol in water): *t*_R_ = 6.14 min, 98.64%.

*3-(2-([1,1′-biphenyl]-4-yl)-2-oxoethylidene)octahydro-4H-pyrido[1,2-a]pyrazin-4-one* (**3f**) Yield 47%. Yellow solid. m.p. 197–199 °C. ^1^H-NMR (300 MHz, DMSO-*d*_6_): δ 10.70 (s, 1H), 7.97 (d, *J* = 8.31 Hz, 2H), 7.80 (d, *J* = 8.31 Hz, 2H), 7.74 (d, *J* = 7.23 Hz, 2H), 7.52 (t, *J* = 7.46 Hz, 2H), 7.45–7.40 (m, 1H), 6.67 (s, 1H), 4.42 (d, *J* = 13.26 Hz, 1H), 3.68–3.63 (m, 2H), 3.34–3.24 (m, 1H), 2.79–2.70 (m, 1H), 1.82–1.79 (m, 3H), 1.52–1.25 (m, 3H). IR (cm^−1^, KBr film): 3415 (-NH), 1659 (-CON), 1604 (-CO). HRMS (ESI): calcd. for C_22_H_23_N_2_O_2_ [M + H]^+^ 347.1754, found 347.1757, HPLC (80% methanol in water): *t*_R_ = 11.36 min, 99.10%.

*3-(2-(furan-2-yl)-2-oxoethylidene)octahydro-4H-pyrido[1,2-a]pyrazin-4-one* (**3g**) Yield 63%. Yellow solid. m.p. 152–154 °C. ^1^H-NMR (300 MHz, DMSO-*d*_6_): δ 10.31 (s, 1H), 7.88–7.87 (m, 1H), 7.15–7.13 (m, 1H), 6.66–6.64 (m, 1H), 6.45 (s, 1H), 4.39 (d, *J* = 13.47 Hz, 1H), 3.67–3.58 (m, 2H), 3.29–3.20 (m, 1H), 2.77–2.68 (m, 1H), 1.80–1.77 (m, 3H), 1.51–1.38 (m, 3H). ^13^C-NMR (300 MHz, CDCl_3_) δ: 180.1, 159.7, 154.0, 150.2, 145.2, 114.2, 112.0, 92.1, 53.8, 44.2, 43.3, 29.9, 24.4, 22.8. IR (cm^−1^, KBr film): 3415 (-NH), 1670 (-CON), 1612 (-CO). HRMS (ESI): calcd. for C_14_H_17_N_2_O_3_ [M + H]^+^ 261.1234, found 261.1234, HPLC (80% methanol in water): *t*_R_ = 4.54 min, 98.87%.

*3-(2-oxo-2-(thiophen-2-yl)ethylidene)octahydro-4H-pyrido[1,2-a]pyrazin-4-one* (**3h**) Yield 70%. Yellow solid. m.p. 182–184 °C. ^1^H-NMR (300 MHz, DMSO-*d*_6_): δ 10.31 (s, 1H), 7.83–7.81 (m, 1H), 7.72–7.71 (m, 1H), 7.20–7.16 (m, 1H), 6.47 (s, 1H), 4.40 (d, *J* = 13.41 Hz, 1H), 3.64–3.58 (m, 2H), 3.28–3.21 (m, 1H), 2.77–2.68 (m, 1H), 1.80–1.77 (m, 3H), 1.52–1.25 (m, 3H). IR (cm^−1^, KBr film): 3422 (-NH), 1673 (-CON), 1601 (-CO). HRMS (ESI): calcd. for C_14_H_17_N_2_O_2_S [M + H]^+^ 277.1005, found 277.1011, HPLC (80% methanol in water): *t*_R_ = 5.13 min, 98.89%.

*3-(2-oxo-2-(thiazol-2-yl)ethylidene)octahydro-4H-pyrido[1,2-a]pyrazin-4-one* (**3i**) Yield 46%. Yellow solid. m.p. 151–153 °C. ^1^H-NMR (300 MHz, DMSO-*d*_6_): δ 10.47 (s, 1H), 8.04–8.02 (m, 2H), 6.88 (s, 1H), 4.38 (d, *J* = 13.44 Hz, 1H), 3.69–3.63 (m, 2H), 3.34–3.26 (m, 1H), 2.79–2.70 (m, 1H), 1.82–1.78 (m, 3H), 1.51–1.25 (m, 3H). IR (cm^−1^, KBr film): 3448 (-NH), 1671 (-CON), 1612 (-CO). HRMS (ESI): calcd. for C_13_H_16_N_3_O_2_S [M + H]^+^ 278.0958, found 278.0961, HPLC (80% methanol in water): *t*_R_ = 4.37 min, 98.28%.

*3-(2-(naphthalen-1-yl)-2-oxoethylidene)octahydro-4H-pyrido[1,2-a]pyrazin-4-one* (**3j**) Yield 50%. Yellow solid. m.p. 151–153 °C. ^1^H-NMR (300 MHz, DMSO-*d*_6_): δ 10.63 (s, 1H), 8.36–8.31 (m, 1H), 8.02–7.95 (m, 2H), 7.69–7.67 (m, 1H), 7.57–7.52 (m, 3H), 6.31 (s, 1H), 4.36 (d, *J* = 13.23 Hz, 1H), 3.71–3.63 (m, 2H), 3.33–3.29 (m, 1H), 2.75–2.67 (m, 1H), 1.81–1.72 (m, 3H), 1.46–1.22 (m, 3H). IR (cm^−1^, KBr film): 3442 (-NH), 1656 (-CON), 1608 (-CO). HRMS (ESI): calcd. for C_20_H_21_N_2_O_2_ [M + H]^+^ 321.1598, found 321.1602, HPLC (80% methanol in water): *t*_R_ = 6.84 min, 96.12%.

### 4.2. Biology

#### 4.2.1. Cell lines and Culture

HepG2-ARE-C8 cells were kindly provided by Professor Rong Hu (China Pharmaceutical University, Nanjing). Cells were maintained in modified RPMI-1640 medium (GiBco, Invitrogen Corp., Carlsbad, CA, USA) with 10% fetal bovine serum (FBS) (GiBco, Invitrogen Corp., Carlsbad, CA, USA) in a humidified atmosphere of 5% CO_2_ and 95% air at 37 °C. HCT116 were cultured in McCoy’s 5A (Sigma-Aldrich, St. Louis, MO, USA) and supplemented with 10% (*v*/*v*) FBS.

#### 4.2.2. Cell Viability Assay

Cell viabilities were determined by using 3-[4,5-dimethylthiazol-2-yl]-2,5-diphenyltetrazolium bromide (MTT) assay. MTT was dissolved in (phosphate buffered solution) PBS to a concentration of 5 mg/mL. HCT116 cells (8 × 10^3^ cells/well) were plated in 96-well plates and incubated overnight. Different concentrations of test compounds were added to each cell for 24 h at 37 °C, and the 20 μL MTT solution was added and incubated for an additional 4 h. Then, 100 μL of DMSO was added to dissolve formazan precipitate after the solution was removed. The OD value was determined at 570 nm by Elx800 absorbance microplate reader (BioTek, Winooski, VT, USA). IC_50_ value (concentration at which cell survival equals 50% of control) = [1 − (OD_test_ − OD_blank_)/(OD_control_ − OD_blank_)] × 100%.

#### 4.2.3. ARE-Luciferase Activity Assay

HepG2-ARE-C8 cells (4 × 10^4^ cells/well) were incubated overnight in 96-well plates, and then treated with different concentrations of test compounds. tBHQ was used as a positive control, DMSO was used as a negative control, and the luciferase cell culture lysis reagent was used as a blank. The medium was removed after 12 h treatment. Then, the cells were harvested in the luciferase cell culture lysis reagent together with 100 μL of cold PBS. Then, centrifuging the solution and 20 μL of the supernatant was used for determining the luciferase activity. The luciferase activity was measured by a Luminoskan Ascent (Thermo Scientific, Waltham, MA, USA). The data were obtained in triplicate and expressed as fold induction over the control. Induction fold = (RLU_test_ − RLU_blank_)/(RLUDMSO_control_ − RLU_blank_). RLU = relative light unit l [20].

#### 4.2.4. Western Blot

Anti-NQO1 (sc-271116), anti-Nrf2 (BS1258) and Anti-β-actin (AP0060) antibodies were purchased from Santa Cruz Biotechnology (Santa Cruz, CA, USA) and Bioworlde (Bioworlde, Visalia, CA, USA), respectively. The isolation of cell fractions and Western blotting were performed as detailed previously [16]. Briefly, the extracts were separated by SDS-PAGE and then electrotransferred to PVDF membranes (Perkin Elmer, Northwalk, CT, USA). Membranes were blocked with 1% BSA for 1 h followed by incubation with a primary antibody at 4 °C overnight. Then they were washed and treated with a DyLight 800 labeled secondary antibody at 37 °C for 2 h. The membranes were screened through the odyssey infrared imaging System (LI-COR, Lincoln, NE, USA).

### 4.3. Physicochemical Properties

The p*K*a and distribution coefficient (log *D*, pH 7.4) were determined according to the methods of Avdeef and Tsinman on a Gemini Profiler instrument (pION) by the “goldstandard” Avdeef-Bucher potentiometric titration method. Permeability (Pe) was determined by a standard parallel artificial membrane permeability assay (PAMPA by pION). The pH-metric method was used to determine the intrinsic solubility. This is a new potentiometric acid−base titration method. The potentiometric solubility data were obtained with the pSOL model 3 instrument (pION INC., Cambridge, MA, USA) and subsequently processed with the accompanying computer program, pS.

## Figures and Tables

**Table 1:** Induction fold of antioxidant response element (ARE) activity in HepG2-ARE-C8 cell and IC_50_ value measured in HCT116 cells of Series I, II and III compounds.

Compound	ARE Inductivity in Luciferase Reporter Assay (μM) ^a^						IC_50_ (μM)
	0.1	1	5	10	50	100	
**1a**	0.56 ± 0.28	1.66 ± 0.05	2.40 ± 1.18	5.64 ± 2.06	7.53 ± 1.28	7.62 ± 2.79	>300
**1b**	1.26 ± 0.28	1.26 ± 0.73	2.78 ± 0.29	3.45 ± 0.03	2.75 ± 0.68	2.68 ± 0.31	>300
**1c**	0.80 ± 0.19	0.78 ± 0.36	2.30 ± 0.39	3.05 ± 0.53	3.09 ± 0.09	3.41 ± 0.65	>300
**1d**	1.24 ± 0.23	1.09 ± 0.72	1.49 ± 0.16	2.94 ± 1.90	7.14 ± 0.34	10.07 ± 0.05	>300
**1e**	1.57 ± 0.05	1.97 ± 0.20	4.53 ± 0.28	5.48 ± 0.13	7.80 ± 3.94	34.95 ± 3.15	183.3
**2a**	2.38 ± 0.11	2.28 ± 0.78	2.55 ± 0.31	3.09 ± 1.09	6.74 ± 0.67	19.60 ± 0.47	>300
**2b**	3.71 ± 1.41	3.57 ± 1.08	4.85 ± 0.82	6.58 ± 2.00	8.24 ± 0.62	12.90 ± 1.73	>300
**2c**	2.96 ± 0.48	3.04 ± 0.08	5.73 ± 1.44	13.35 ± 0.44	27.96 ± 1.39	29.01 ± 2.45	190.3
**2d**	1.06 ± 0.09	3.19 ± 0.52	4.06 ± 0.30	7.38 ± 0.31	5.22 ± 1.28	5.83 ± 0.38	160.8
**2e**	1.92 ± 0.82	2.17 ± 1.05	3.03 ± 0.68	3.49 ± 0.64	3.92 ± 0.53	8.31 ± 0.25	>300
**2f**	3.27 ± 1.59	2.10 ± 0.59	5.46 ± 1.83	8.45 ± 1.05	3.34 ± 1.05	3.07 ± 0.43	170.7
**2g**	1.27 ± 0.52	2.31 ± 0.24	4.68 ± 0.29	5.84 ± 0.07	14.47 ± 0.36	9.69 ± 0.80	>300
**2h**	2.06 ± 0.98	3.40 ± 0.02	3.62 ± 0.29	4.59 ± 0.14	4.38 ± 1.94	4.98 ± 0.53	92.36
**2i**	1.71 ± 0.71	1.88 ± 1.36	1.37 ± 0.19	1.66 ± 0.53	1.16 ± 0.63	1.42 ± 0.31	237.0
**3a**	1.20 ± 0.49	4.09 ± 0.79	15.85 ± 2.08	16.38 ± 0.55	29.90 ± 8.49	40.60 ± 1.78	>300
**3b**	0.70 ± 0.35	5.98 ± 2.31	24.78 ± 2.67	32.97 ± 3.68	39.97 ± 0.69	4.41 ± 2.89	168.5
**3c**	1.85 ± 0.21	8.41 ± 0.04	10.19 ± 3.44	11.41 ± 1.12	1.13 ± 1.03	1.33 ± 0.19	174.3
**3d**	1.96 ± 0.88	3.85 ± 1.00	12.02 ± 2.55	19.52 ± 3.09	47.58 ± 3.54	8.66 ± 3.27	180.8
**3e**	1.40 ± 1.21	1.82 ± 1.15	6.59 ± 0.77	9.16 ± 0.42	9.36 ± 3.76	1.44 ± 1.75	>300
**3f**	2.96 ± 0.66	2.21 ± 0.52	3.60 ± 0.42	1.76 ± 0.10	2.47 ± 0.03	2.69 ± 2.75	183.7
**3g**	0.24 ± 0.15	1.50 ± 0.01	6.41 ± 3.14	27.01 ± 2.46	41.61 ± 2.60	79.74 ± 1.92	>300
**3h**	2.06 ± 0.22	2.12 ± 0.31	8.30 ± 2.55	10.52 ± 3.82	28.93 ± 0.46	1.63 ± 0.25	>300
**3i**	1.52 ± 0.87	4.02 ± 3.05	6.41 ± 1.48	9.28 ± 0.55	13.54 ± 0.80	16.74 ± 1.39	>300
**3j**	1.7 ± 0.19	2.34 ± 0.53	2.54 ± 0.99	3.52 ± 0.57	3.85 ± 0.34	1.96 ± 0.71	263.5
**2**	1.45 ± 0.39	6.20 ± 4.06	16.19 ± 0.30	31.17 ± 4.14	7.51 ± 1.32	12.16 ± 3.32	165.1
tBHQ	1.00 ± 0.01	1.80 ± 0.66	1.67 ± 0.56	1.98 ± 0.29	4.45 ± 1.15	10.79 ± 3.13	ND
DMF	1.04 ± 0.21	1.10 ± 0.11	1.40 ± 0.21	2.06 ± 0.25	3.34 ± 0.52	5.31 ± 0.24	ND

^a^ Values shown are mean ± SD (*n* = 3, 12 h of treatment). Cells were exposed to compounds tested for their ability to increase levels of relative luciferase units.

**Table 2:** Physicochemical properties of selected compounds.

Compound	p*K*a	log *D*, pH 7.4	Pe (10^−6^ cm/s)	Solubility (mg/mL)
			pH 7.4	
**1e**	2.90	0.61	52.954 ± 7.290	0.046
**2c**	3.02	1.22	43.953 ± 5.421	0.17
**3a**	3.42	0.98	66.372 ± 5.082	0.53
**3b**	3.10	1.23	54.550 ± 6.001	0.29
**3g**	2.08	−0.34	32.712 ± 1.922	3.31
**2**	2.78	2.38	11.680 ± 2.198	0.022
Ketoprofen	-	-	1.607 ± 0.243	-
Propranolol	-	-	64.679 ± 8.582	-
